# Cognitive Impairment After COVID-19—A Review on Objective Test Data

**DOI:** 10.3389/fneur.2021.699582

**Published:** 2021-07-29

**Authors:** Rania Daroische, Mathilde S. Hemminghyth, Thomas H. Eilertsen, Monica H. Breitve, Luiza J. Chwiszczuk

**Affiliations:** ^1^Neuropsychological Unit, Helse-Fonna HF Haugesund Hospital, Haugesund, Norway; ^2^Department of Research and Innovation, Helse-Fonna HF Haugesund Hospital, Haugesund, Norway; ^3^Department of Geriatric Psychiatry, Clinic of Psychiatry, Helse-Fonna HF Haugesund Hospital, Haugesund, Norway

**Keywords:** COVID-19, coronavirus, SARS-CoV-2, cognitive impairment, cognitive function, neuropsychology

## Abstract

**Objective:** The aim was to conduct a review on the literature on objective cognitive impairment in patients after COVID-19.

**Methods:** We performed a literature review and searched Ovid Medline in February 2021 based on a PECO scheme.

**Results:** Twelve articles met all inclusion criteria. Total patient sample was <1,000. All studies on global cognitive function found impairment, ranging from 15 to 80% of the sampled patients. Seven studies on attention and executive functions reported impairment, with varying results depending on sub-domain and different tests. Three out of four studies reported memory difficulties, with two studies reporting short-term memory deficits. Although results indicate possible language impairment, only one study used domain-specific language tasks. Two out of four studies on visuospatial function did not report any impairment.

**Conclusion:** Patients with recent SARS-CoV-2 infection appear to experience global cognitive impairment, impairment in memory, attention and executive function, and in particular verbal fluency. Based on the current results, we recommend clinicians to evaluate the need for cognitive assessment of patients with a recent COVID-19 infection, regardless of the severity of the disease, treatment methods and length of ICU stay. We need studies with larger sample and control group.

## Introduction

Severe acute respiratory syndrome coronavirus 2 (SARS-CoV-2) causes the coronavirus disease 19 (COVID-19). It first appeared in Wuhan, Hubei, China (1), and then spread to the rest of the world, making it a pandemic. The virus belongs to the *Coronaviride* family. Over the past 10 years, there have been two other coronavirus epidemics that caused severe infections: the severe acute respiratory syndrome (SARS-CoV) epidemic in 2003 (2) and the Middle East respiratory syndrome (MERS-CoV) (3). COVID-19 was reported to be primarily a lower respiratory tract disease, and common symptoms included fever, cough, and shortness of breath (1). At the same time, the severity varies, ranging from asymptomatic or very mild symptoms, such as a cold or pneumonia, to very severe symptoms and acute respiratory failure insufficiency (4).

Reports about anosmia (loss of the sense of smell) (5) and ageusia (loss of taste) in patients with COVID-19 infection turned attention toward possible affection of the central nervous system (CNS) (6–9). Other early complications include impaired consciousness, agitation, dizziness and headache (7). After recovery, fatigue, anxiety, depression, insomnia have also been reported as common symptoms (10). Rogers and colleagues (11) conducted a systematic review and found a few studies that did systematic assessments of cognition in patients following SARS-CoV and MERS-CoV infection. During acute phase, around a third of the patients experienced impaired memory, concentration or attention (11). After the illness, around one fifth of all patients had one or more of the aforementioned cognitive impairments. A letter dating from June 2020 (12) reported that a third of their discharged COVID-19 patients showed a dysexecutive syndrome consisting of “inattention, disorientation, or poorly organized movements in response to command” (Third paragraph). As more unusual symptoms emerged it became gradually clear that COVID-19 could affect a wide variety of organs and tissue (13–15).

The etiology of the SARS-CoV-2 is certainly multifactorial, but the exact pathophysiological mechanisms leading to the neurological and psychiatric consequences of COVID-19 is still not clear. In earlier coronavirus infections, the following neurotoxic mechanisms are described:

1. Neurotropism and direct ability to enter neurons and glial cells, leading to neuronal dysfunction and damage (neuroinvasion), and secondly to i.e., encephalitis. The virus may reach the CNS indirectly through the blood-brain barrier and/or directly by axonal transmission across olfactory neurons (16–20).
2. Affection of cerebral blood vessels and coagulopathies causing ischemic or hemorrhagic strokes (21–23).
3. Secondary negative consequences of excessive systemic inflammatory responses, “cytokine storm” and peripheral organ dysfunctions affecting the brain (24, 25).
4. Global ischemia secondary to respiratory insufficiency, respirator treatment and so-called acute respiratory distress syndrome ARDS (26).

Heneka et al. (48) suggest that all the previously mentioned mechanisms could play a role in the etiology of the cognitive impairment in COVID-19 survivors. There is an ongoing discussion whether COVID-19 may cause long-term cognitive impairments. Such a theory is supported by several studies showing a link between infections with human herpes viruses and the risk of dementia development later in life (27). Neurodegeneration could possibly emerge many years after viral infections in the CNS, which some considers was the case in encephalitis lethargica, where extrapyramidal symptoms emerged long after recovery of Spanish influenza in 1918 (28). Evidence shows that inflammation is a risk factor for persistent cognitive decline in survivors of ARDS or sepsis (29, 30). In addition, high cytokine levels during ≪cytokine storm≫ predicts the occurrence of hippocampal atrophy (31).

There have been published a few reviews on cognitive impairment after COVID-19 infection, however none of them are—to our best knowledge–solely based on objective neuropsychological data. As there are no clear link between subjective reports of impaired cognition and findings on objective tests (32), we chose to only include studies that reported objective test data. It is important to report and address specific cognitive impairments after COVID-19, as this enables us to adapt current and/or implement new rehabilitation programs. Early identification and properly tailored rehabilitation will most likely reduce negative health effects and address the socio-economic consequences at a population level.

## Methods

### Search Strategy

We formulated the search strategy based on PECO scheme (Population/problem; Exposure, Comparison; Outcome) and searched Ovid MEDLINE for studies performed between January 2019 to 16th February 2021. The purpose was to identify human studies on objective cognitive and neuropsychological consequences of COVID-19 in recovered adult patients.

For Medline MESH terms and keywords, derivations and combination of them were used: [(COVID-19 OR sars-cov-2 OR Betacoronavirus/ OR Betacoronavirus OR Coronavirus Infections/ OR corona OR Coronavirus) AND (cognit^*^ OR neuropsycholog^*^)]. The search was restricted to references published in 2019 up until February 2021 and performed in collaboration with a university librarian. References from reviewed articles were also searched for relevant studies.

After removing all duplicates electronically using EndNote, we removed studies that was not written in English. The search results were then divided equally among all authors to first screen titles and abstracts where we removed articles with irrelevant topics and articles without objective cognitive testing.

After preliminary selection, two of the authors (R.D. and L.J.C.) independently reviewed all previously selected titles and abstracts. In case of uncertainty as to whether a given article should be included in the review, all authors assessed the entire article and was then discussed in plenum until consensus was achieved.

### Selection Criteria

#### Inclusion Criteria

We included all studies that assessed cognition with the use of at least one neuropsychological instrument or test. They had to report either patients scoring below or above a set of cut-off, or the exact test score. The assessment had to be performed directly with the patients, with remote tablet applications or by telephone or video calls after the acute phase of infection. Participants had to fulfill the WHO clinical criteria of COVID-19 or have a laboratory confirmed infection. We included cohort studies, cross sectional studies, and letters if they included original research with relevant data.

#### Exclusion Criteria

We excluded case reports and case series as we aimed to provide a summary of quantitative data. We also excluded studies where direct neurological complications, such as ischemic or hemorrhagic stroke, delirium and acute encephalopathies, caused the cognitive impairment, and where patients had known pre-morbid mild cognitive impairment (MCI) or dementia. We also excluded studies that was not yet peer-reviewed. This literature review follow AMSTAR 2 guidelines.

## Results

**Figure 1:**
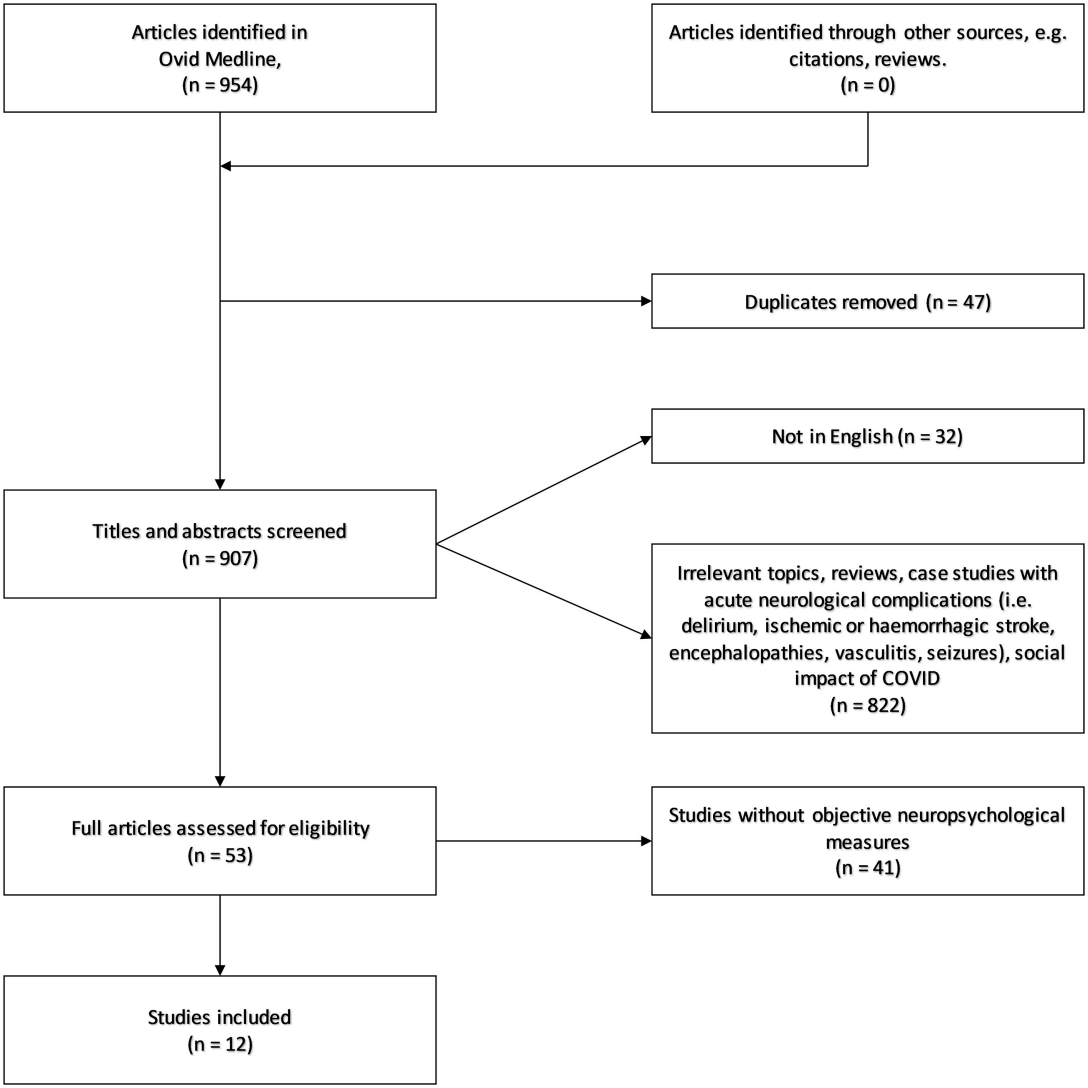
Flow chart for inclusion and exclusion of studies.

The search initially yielded 954 references. Of those, we identified and removed 47 duplicates and 32 articles in a language other than English. Of the remaining 875 studies, 822 were removed based on title and abstract screen. We assessed full text for 53 articles, and ended up with 12 included studies. See Figure 1 for flow chart.

### Findings

The patients completed testing and examination ranging from a few days after symptom onset (33) to several months after hospital discharge (34).

There are differences in how the studies were conducted, as shown for time of assessment. This makes it not possible to conduct a proper analysis at a more general level. Similarly, so few studies used the same methodology, analyzing and comparing them would result in low power, and a high probability of false negatives. Hence, we would be unable to know whether the variation (or lack thereof) is due to patients being tested early vs. late (e.g., patients in an acute phase might display temporary symptoms; temporary conditions, such as reduced general condition, might mask cognitive difficulties; cognitive difficulties might be more pronounced during an early phase, and regress later on).

The range of included participants was nine (35) to 185 (36). Researchers mostly studied patients in Europe (9), South America (2) and China (1). In majority of the studies (*n* = 9) more than half of the participants were male. Furthermore, the researchers tested the patients by telephone, video calls, remote tablet applications (*n* = 4) or directly with the patients (*n* = 7). In addition, one study used both methods (remote and beside). The time from the acute phase of the COVID infection varied from a few days (37) to 6 months (38). There is also a considerable variation in the severity of the COVID-19 disease, where some studies assessed patients who had been critically ill and needed mechanical ventilation for several days (34, 37) to others assessing outpatients who had only mild symptoms (38). See Table 1 for an overview of descriptive data and results for included studies on cognitive manifestations of COVID-19.

**Table 1:** Overview of descriptive data and results for included studies on cognitive manifestations of COVID-19.

**Authors**	**Country**	***N*/% females**	**Age *M* ±*SD***	**Assessment method**	**Assessment time**	**Mechanical ventilation treatment (% of sample)**	**Results**
van den Borst et al. (41)	the Netherlands	124/40%	59.0 ± 14.0	Telephone	3~ months post recovery	16%	15% of patients scored <34 on TICS
Beaud et al. (37)	Switzerland	13/23%	64.8 ± 7.6	Bedside	5.5 ± 2.4 days post ICU discharge	100%	MoCA *M* = 19.7 ± 7.5 FAB *M* = 10.9 ± 5.5
De Lorenzo et al. (36)	Italy	185/34%	57 (median)	Outpatient	Median 23 days (range 20–29) post discharge	17 %	25.4% of patients scored <24 on MoCA
Zhou et al. (44)	China	29/38%	47.0 ± 10.5	iPad based online	Usually 2–3 weeks post infenction	N/A^c^	No difference between patients and controls for TMT, SDT and DST. Covid-19 patients performed significantly worse than control on parts of CPT, especially correct and missed responses when facing increased demands on sustained attention.
Almeria et al. (42)^a^	Spain	35/54%	47.6 ± 8.9	Inpatient	10–35 post discharge	N/A	Impaired scores (>2SD below norms) for 2.9–11.4% of patients on TAVEC, DST backwards, TMT-A, TMT-B, SDMT, Stroop color, Stroop interference, Semantic fluency, Phonemic fluency, WMS-IV: VR, BNT.
Woo et al. (43)	Germany	18/57.9%	Mean age (range) 42.11 (17–71).	Either by phone or directly with the patient	20–105 days after recovery	33.3%	Post-COVID-19 patients scored significantly lower results in the TICS-M (mean, 38.83; range, 31–46) compared to healthy controls (mean, 45.8; range, 43–50), especially regarding short-term memory, attention and concentration/language tasks.
Negrini et al. (35)^a^^b^	Italy	9/33%	60.4 ± 16.3	Tablet-supported video calls	1~ month post hospitalization	56%	MMSE *M* = 26.5 ± 2.9 FAB *M* = 15.4 ± 2.3
Del Brutto et al. (38)	Equador	52/62%	59.4 ± 10.6	Outpatient	6 months after the start of the SARS-CoV-2 pandemic in the village	0%	Cognitive decline in 11 of 52 (21%) with a history of mild symptomatic SARS-CoV-2 infection, on MoCA.
Alemanno et al. (33)	Italy	87/28.74%56 at follow up	67.23 ± 12.89	Inpatient and 1 month after hospital discharge	Five to 20 days after symptoms onset	35.6% with orotracheal intubation and ventilation from 1–27 days20.6% with non-invasive ventilation (NIV) using Biphasic Positive Airway Pressure.33.3% who received oxygen therapy with Venturi Masks or reservoir Masks.	Group 1 (Orotracheal intubation): MoCA *M* = 21.65 ± 5.23; 54.5% had deficits at FUP MMSE *M* = 26.77 ± 2.77; 9.1% had deficits at FUP Group2 (Non-invasive ventilation): MoCA *M* = 16.83 ± 7.11; 83.3% had deficits at FUP MMSE *M* = 22.78 ± 5.80; 8.3% had deficits at FUP Group 3 (Venturi Masks): MoCA *M* = 15.90 ± 6.97; 85% had deficits at FUP MMSE *M* = 22.24 ± 6.23; 35% had deficits at FUP Group 4 (No oxygen therapy): MoCA *M* = 19.11 ± 6.83; 100% had deficits at FUP MMSE *M* = 22.89 ± 6.97; 50% had deficits at FUP
Soldati et al. (34)	Brazil	23/21.73%	53.6 ± 11.7	Telephone	Median 83 days post discharge for first contact, then 15 days for testing	100%	TICS *M* = 31.9 ± 1.2 39% of patients scored <33 on TICS
Ortelli et al. (39)	Italy	12/16.67%	67 ± 9.6	Inpatient rehabilitation, laboratory setting	Median: 12 weeks (range: 9–13)	N/A	Cognitive deficits, particularly executive deficits. Significantly poorer global MoCA and FAB scores as compared to HCs, mean 17.8 and 12.2, respectively. In regards to vigilance and executive attention, patients had significantly longer RTs in two out of three computerized tasks, while the error percentage was significantly higher in all three tasks compared to HCs.
Raman et al. (40)	United Kingdom	58/41.4%	55.4 ± 13	Outpatient	Median: 1.6 months after discharge (IQR: 1.4–1.8)	36.2%	Impaired executive/visuospatial function: MoCA-score < =4 in 40 vs. 16 % of HCs. 28% had a total MoCA-score under cut-off (<26) compared to 17% of HCs.

*TICS, Telephone interview of Cognitive Status; FAB, Frontal Assessment Battery; MoCA, Montreal Cognitive Assessment; TMT, Trail Making Test; SDT, Sign Coding Test; DST, Digital Span Test; CPT, Continuous Performance Test; SDMT, Symbol Digit Modalities Test; BNT, Boston Naming Test; TAVEC, Test de Aprendizaje Verbal España-Complutense; WMS-IV: VR, Wechsler Memory Scale – Visual reproduction, MMSE, Mini-Mental State Examination*.

a *Both studies are case series*.

b *Negrini et al. (35) corrected raw scores for age, sex and education*.

Six studies used Montreal Cognitive Assessment (MoCA) (33, 35, 37, 39, 40), one study (33) assessed global cognition with Mini Mental State Examination (MMSE), and two studies used Telephone Interview for Cognitive Status (TICS) (34, 41). All of the abovementioned studies used generally accepted cut-off scores for defining cognitive impairment, while Almeria and colleagues (42) created a cognitive index score by obtaining an arithmetic mean of the standardized scores of the different cognitive test.

#### Global Cognitive Function

Among the studies employing a measure of global cognitive function, all of them found cognitive impairment in some, but not all of the patients. The percentage of patients with global cognitive impairment ranged from 15% in Van Den Borst et al. (41), to 80% in Alemanno et al. (33). MoCA was the most common test for assessing global cognitive function, and among those reporting mean and standard deviations for scores, the results varied from 15.90 ± 6.97 in patients with Venturi mask (33), to 26.50 ± 2.90 in patients 1 month after hospitalization (35). In the largest study on global cognitive function, 25.4% of the 185 patients (36) had cognitive impairment, defined as a total MoCA score of <24 points 3–4 weeks after discharge from ICU. Raman et al. (40) found that 28% of the patients had a total MoCA score under the established cut-off of <26 compared to 17% (5/30) of the controls. However, the median MoCA scores in patients were not significantly different from that of controls (40). Comparing patients to healthy controls, Ortelli and colleagues (39) found that the average score was significantly lower for the patient sample (17.8 vs. 26.8 on MoCA). Using both MoCA and MMSE, Alemanno and colleagues (33) classified 19.6% of patients as having cognitive deficits based on total MMSE scores, and 73.2% based on total MoCA scores. They also reported higher scores in younger patients and interestingly, in those that had the most aggressive oxygen/respiratory therapy.

#### Attention and Executive Function

Seven studies assessed attention or executive function, either by using sub-test scores of global cognitive measures (MMSE, MoCA) or by using more advanced and specific neuropsychological measures (i.e., computerized attentive tasks). Interestingly, all of the studies found some executive or attentional deficit (35, 37, 39, 40, 42–44), as well as all of the studies that investigated executive function also found some attentional deficit (33, 35, 37, 39, 42–44). Raman et al. (40) did however not specify the results in the attentional domain.

Almeria et al. (42) only found one patient (2.9%) to have a processing speed (Trail Making Test A) and inhibition impairment (Stroop Interference), while two patients (5.7%) had pathological scores in a test that measures divided attention and visual scanning (Symbol Digit Modalities Test, SDMT). Three participants (8.6%) had difficulties with cognitive flexibility (Trail Making B) and attention span (Inverse Digits).

Three of the studies tested patients with Frontal Assessment Battery (FAB) (35, 37, 39), which assess different aspects of executive functions, like fluency, inhibition, conceptualization and more. All the three studies found abnormal executive scores, in varying degrees. Negrini et al. (35) only found abnormal scores in one patient (11.1%), while Beaud et al. (37) found abnormal scores in 8/13 patients (61%).

Four studies found impairments in fluency and language tasks. Beaud et al. (37) found deficits with lexical fluency, while both Negrini et al. (35) and Almeria et al. (42) found pathological phonemic fluency scores in 11% of patients. However, when it comes to semantic fluency, Almeria et al. (42) only found that 5.7% had impairment.

#### Memory

Four studies included cognitive tests of memory (33, 35, 37, 42, 44) whereas three of them found impairments (33, 35, 37, 42). Almeria et al. (42) only found pathological score in one patient (2.9%) on a verbal memory test (Tavec Total), while two other studies reported deficits in short-term memory (33, 35, 43).

#### Language

Five patients (16.7%) in the study with Woo et al. (43) experienced subjective reduction in words findings and this found objective confirmation in language tasks from TICS to investigate the language domain. The authors found significant reduced scores in concentration/language tasks, compared to healthy controls but did not provide detailed number Only one study investigated domain-specific language tasks (42) and found that one patient had lower scores in naming test (Boston naming test).

#### Visuospatial Function

Four studies investigated visuospatial function (37, 42, 44). Beaud et al. (37) found impairments in visuospatial functions when testing with MoCA, while Raman et al. (40) found that the visuospatial domain was impaired for 40% of patients with COVID-19, compared to 16% of the control group, when testing with MoCA. Zhou et al. (44) did not find a significant difference between patients with COVID-19 and the control group when testing with Trail Making B. Almeria et al. (42) did not find abnormalities, when testing Visual Reproduction with the Wechsler Memory Scale and visuospatial organization (Rey-Osterrieth Complex test—copy).

## Discussion

Based on the reported evidence it seems that patients in various degrees, suffer from short-term cognitive impairment following COVID-19 infection. Compared to healthy controls, all of the included studies reported that a higher percentage of patients had a global cognitive impairment. In regards to specific cognitive domains, principally attentional and executive functions seems to be prone to impairments. The data on memory, language and visuospatial functions are on the other hand less reliable.

The latter is possibly related to observed heterogeneity in methods, e.g., what type of instrument or test they used, time of assessment (early vs. late in the disease process), inclusion and exclusion criteria and to what extent they reported precise data. Although little test data is reported from the related SARS-CoV or Mers CoV infections (45), Rogers and colleagues (11) did indeed report that even though a third of patients experienced cognitive difficulties in the early phase, only one fifth of patients had cognitive impairments at a later stage. At the same time, this would be somewhat in accordance with one fourth of the COVID-19 patients in De Lorenzo et al. (36) showing cognitive impairment after discharge. In order to analyze time gradients for cognitive impairment following COVID-19 infections one would require more research with similar methods.

The results of a large, internet-based study, including 84 285 participants who recovered from the COVID-19, suggests a higher incidence of cognitive dysfunction compared to controls (46). The authors found that COVID-19 infection may lead to cognitive impairment, and that the impairment is likely to affect multiple cognitive domains. Unfortunately, only 361 of the participants had laboratory confirmed COVID-19 infection and the study was not yet peer-reviewed by the time of the search, and therefore not included in our review. As of this date, there are only a few studies on the objective short-term cognitive consequences of COVID-19 with all together <1,000 patients was included. Reported results for cognitive impairment ranged from as low as 15% (41) to 80% of the patients (33). Although using the same measurement, differences in assessment time (i.e., different “stages” of the disease) could explain some of the variation. E.g., Negrini et al. (35) assessed their patients 1 month post hospitalization compared to Beaud et al. (37) that used bedside assessments.

The most common global cognitive screening tool administered in the studies was MoCA (*n* = 6), followed by TICS or TICS-M (*n* = 3) and MMSE (*n* = 2). Comparing patient populations using different tools, each with different sensitivity and specificity, might explain a huge range in the estimates. E.g., Alemanno et al. (33) used both MoCA and MMSE and found that patients had partly recovered their cognitive impairment at 1-month follow up when tested with MMSE. However, using the accepted cut-off on MoCA, most of these patients would still be classified as cognitively impaired. This would be in line with a meta-analysis on the diagnostic ability of MMSE and MoCA for detecting mild cognitive impairment in the elderly. Ciesielska et al. (49) found sensitivity around 66 and 80%, and specificity around 73 and 81% for MMSE and MoCA, respectively. This point to a related need, namely the need for employing test batteries encompassing multiple cognitive domains. One such study for COVID-19 (42), reported that while <12% of the sample had pathological scores on one given test, around one third of the patients scored below cut-off on any of the tests. If clinicians were to use sub-optimal testing strategies to examine patients' cognitive difficulties, it may lead them to underestimate the need for rehabilitation or follow-up. Considering a study on 75 adult patients with acquired brain injury (47) found improvements in mental health and quality of life following neuropsychological rehabilitation. Hence, lack of such rehabilitation for patients following COVID-19 infection might have similar consequences and negative impact on patients' lives.

Furthermore, some of the studies did not define their cut-off scores, while other chose not to use any, due to the poor validity of the established cut-offs in poorly educated population (38). Del Brutto et al. (38) had, by coincidence, a pre-pandemic cognitive assessment, making them able to have a pre-post design. They found a reduction in MoCA scores between pre- and post-pandemic that was twice as large as it was in the two pre-pandemic MoCA assessments, in individuals with mild symptomatic infection (38). The largest study did on the other hand use a dichotomous screening (36), making it difficult to detect subtle cognitive changes. These differences in how the studies defined cognitive decline is therefore a large issue in the interpretation of their finding.

Some of the studies used control groups to compare the results, which might stand for another part of the variance. Three studies used healthy controls and found cognitive impairment in COVID-19 patients compared to controls (39, 40, 43). Another aspect when trying to summarize the findings is that one should be aware that several of the studies had few subjects included, median 35, and 1/3 had under 20 participants. Furthermore, the studies also varied when it comes to the severity of the COVID-19 infection (from mild to severe), length of stay in the intensive care unit (ICU) or time from COVID-19 to first tests (from days to months). Some of the studies tested patients with mild SARS-CoV-2 infection, while other had a severe infection and need for hospitalization. However, Van den Borst et al. (41) did not find that the severity degree had an association with cognitive status when comparing mild disease with moderate-to-critical disease. On the other hand, Del Brutto et al. (38) found that people with mild symptomatic SARS-CoV-2 infection had 18 times likelihoods of developing cognitive impairment, than individuals without serological shown infection, even without severe illness during acute disease. Soldati et al. (34) tested patients with severe COVID-19 and found that the majority (60%) had normal cognitive results. Two of the studies did not find a correlation on the length of stay in the ICU and cognitive impairment (37, 43), thus implying that cognitive impairment are independent from hospitalization time. However, Negrini et al. (35) found that cognitive decay appeared to be linearly associated with the length of stay in the ICU. Van den Borst et al. (41) who studied patients at an average of 3 months after recovery found cognitive impairment in 15% of the patients, while Del Brutto et al. (38) who approximately studied patients 6 months after the start of SARS-CoV-2 pandemic found lower cognitive functions in 21% of patients with mild infection. This may suggest that patients with COVID-19 may experience long-term cognitive impairment after the infection. Hence, there is a large inconsistency in the findings regarding COVID-19's cognitive sequelae.

There is also differences in the groups with mechanical ventilation and passive oxygen supply. On the one hand, Beaud et al. (37) did not find a correlation between cognitive scores and duration of mechanical ventilation or delay between ICU discharge and cognitive assessment. Furthermore, Woo et al. (43) did not find treatments like oxygen supplementations and drugs, such as remdesivir, tocilizumab or antibiotics, to predict observed cognitive deficits. However, Almeria et al. (42) found that the need for oxygen therapy and diarrhea were associated with impairment in memory, attention and executive functions. On the other hand, Alemanno et al. (33) demonstrated that patients in the most critical clinical state actually were the ones with less reduction in cognitive functions. They found also that the patients who benefited from orotracheal intubation and ventilation had significantly better scores in the attentional and calculation domain compared to patients who received oxygen therapy with Venturi Masks or reservoir Masks (33). This paradoxically implies that patients, who profited from the most aggressive respiratory assistance, had better preserved cognitive functions. However, these patients were also the youngest ones. One can therefore not conclude with certainty on how mechanical ventilation and oxygen supply affects cognitive function in patients with COVID-19. Based on these results, there is also reason to believe that it is important to ensure cognitive assessment of patients with COVID-19 infections regardless of severity degree, treatment methods and length of stay in the ICU.

Another weakness is that the studies has not consistently reported data about gender, age and education. Even though Beaud et al. (37) did not find a correlation between cognitive decline and gender, they did find that age correlated with poorer outcome on assessment with FAB, but not MoCA. Alemanno et al. (33) found that cognitive impairment is mostly correlated with patient's age, while Woo et al. (43) did not find gender or age to explain any difference in cognitive functioning. Future studies is therefore needed, to establish the relationship of cognitive impairment and possible demographic risk factors.

### Study Limitations and Strengths

There are limitations to this review. We only searched the Ovid Medline database, and the number of included studies is low. However, we manually searched all references for potential useful articles that were not included in Ovid Medline and did not find any. We resigned from searching in databases without peer-review articles and preprints. We are aware of the fact that research on COVID-19 and cognition are evolving fast, but we still wanted to base this review on approved data.

Nevertheless, the strength of this review is that this is, to our best knowledge, the first one on objective cognitive impairments related to COVID-19. We therefore had the opportunity to investigate whether there was an objective cognitive decline associated with COVID-19, as opposed to a subjective experience of cognitive change. This review is therefore a good basis for future studies aiming to further investigate cognition in COVID-19.

## Conclusion

The results from this review suggest that patients with recent SARS-CoV-2 infection may experience global cognitive impairment, and often a reduction in attention and executive functions. This indicates that several patients with COVID-19 might benefit from early and tailored neuropsychological rehabilitation. It is however necessary to conduct further research using prospective and controlled study designs and standardized assessment tools. It can be of help to study more detailed medical and social consequences of a pandemic, to be better able to plan treatment and rehabilitation for these patients. Lastly, good and reliable data is also needed to investigate the longer-term consequences of COVID-19 infection.

## Author Contributions

RD conceived the study, and was responsible for drafting and revising the work. All authors participated during the drafting process, study design, data collection, analysis and interpretation, and approved the final version to be published.

## Conflict of Interest

The authors declare that the research was conducted in the absence of any commercial or financial relationships that could be construed as a potential conflict of interest.

## Publisher's Note

All claims expressed in this article are solely those of the authors and do not necessarily represent those of their affiliated organizations, or those of the publisher, the editors and the reviewers. Any product that may be evaluated in this article, or claim that may be made by its manufacturer, is not guaranteed or endorsed by the publisher.
